# Engineering Bafilomycin High-Producers by Manipulating Regulatory and Biosynthetic Genes in the Marine Bacterium *Streptomyces lohii*

**DOI:** 10.3390/md19010029

**Published:** 2021-01-11

**Authors:** Zhong Li, Shuai Li, Lei Du, Xingwang Zhang, Yuanyuan Jiang, Wenhua Liu, Wei Zhang, Shengying Li

**Affiliations:** 1 State Key Laboratory of Microbial Technology, Shandong University, Qingdao 266237, China; lizhong@qibebt.ac.cn (Z.L.); sdu_lishuai@mail.sdu.edu.cn (S.L.); lei.du@sdu.edu.cn (L.D.); zhangxingwang@sdu.edu.cn (X.Z.); jiangyy@qibebt.ac.cn (Y.J.); liuwh@sdu.edu.cn (W.L.); zhang_wei@sdu.edu.cn (W.Z.); 2 Shandong Provincial Key Laboratory of Synthetic Biology, CAS Key Laboratory of Biofuels at Qingdao Institute of Bioenergy and Bioprocess Technology, Chinese Academy of Sciences, Qingdao 266101, China; 3 College of Life Sciences, University of Chinese Academy of Sciences, Beijing 100049, China; 4 Laboratory for Marine Biology and Biotechnology, Qingdao National Laboratory for Marine Science and Technology, Qingdao 266237, China

**Keywords:** *Streptomyces*, bafilomycin, regulatory gene, biosynthesis, fermentation optimization

## Abstract

Bafilomycin A_1_ is the representative compound of the plecomacrolide natural product family. This 16-membered ring plecomacrolide has potent antifungal and vacuolar H^+^-ATPase inhibitory activities. In our previous work, we identified a bafilomycin biosynthetic gene cluster (*baf*) from the marine bacterium *Streptomyces lohii* ATCC BAA-1276, wherein a *luxR* family regulatory gene *orf1* and an *afsR* family regulatory gene *bafG* were revealed based on bioinformatics analysis. In this study, the positive regulatory roles of *orf1* and *bafG* for bafilomycin biosynthesis are characterized through gene inactivation and overexpression. Compared to the wild-type *S. lohii* strain, the knockout of either *orf1* or *bafG* completely abolished the production of bafilomycins. The overexpression of *orf1* or *bafG* led to 1.3- and 0.5-fold increased production of bafilomycins, respectively. A genetically engineered *S. lohii* strain (SLO-08) with *orf1* overexpression and inactivation of the biosynthetic genes *orf2* and *orf3*, solely produced bafilomycin A_1_ with the titer of 535.1 ± 25.0 mg/L in an optimized fermentation medium in shaking flasks. This recombinant strain holds considerable application potential in large-scale production of bafilomycin A_1_ for new drug development.

## 1. Introduction

**Figure 1:**
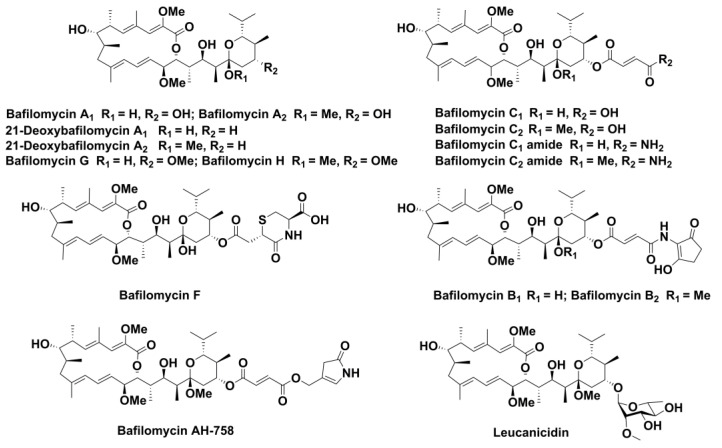
Structures of representative bafilomycins.

Bafilomycins, which are mainly produced by *Streptomyces*, belong to the plecomacrolide (i.e., a 16- or 18-membered macrolactone connected to a 6-membered hemiacetal ring via a three-carbon linker) subfamily of polyketide natural products. These 16-membered ring macrolides have shown diverse biological activities including antibacterial [1], antifungal [2], antitumor [3], and anti-osteoporotic [4] activities. Since bafilomycin A_1_, B_1_, and C_1_ were first isolated from *Streptomyces griseus* sp. sulphurus (TÜ 1922) in 1983 [5], nearly thirty bafilomycin derivatives have been discovered [5,6,7]. Essentially, most of these derivatives (Figure 1) are generated from the bafilomycin A_1_ core structure through various known [8,9,10] and unknown tailoring steps during their biosynthetic pathways.

As the first and archetypal compound of bafilomycins, bafilomycin A_1_ has attracted much attention because it is a potent and specific inhibitor of vacuolar H^+^-ATPase (V-ATPase), which is an important drug target for osteoporosis [4]. This compound may also be applied in antitumor therapy due to its potent autophagy inhibitory activity, which prevents autophagosome-lysosome fusion in cells by targeting the sarco/endoplasmic reticulum Ca^2+^-ATPase (SERCA) pump [11]. Moreover, bafilomycin A_1_ has been demonstrated to show promising prospects in the field of combined pharmacotherapy. For example, bafilomycin A_1_ and FK506 have displayed marked synergistic antifungal activities against the fungal pathogen *Cryptococcus neoformans* [12]; the combined treatment with bortezomib plus bafilomycin A_1_ has been proved to be capable of enhancing the cytocidal effect and inducing U266 myeloma cells [13]; and the inhibition of autophagy by bafilomycin A_1_ can decrease the resistance of gastric cancer cells to 5-fluorouracil in vitro [14]. Intriguingly, bafilomycin A_1_ was recently reported to be capable of interrupting the function of the viral receptor ACE2 via inhibiting the V-ATPase, thus being considered as a candidate for treating the infections caused by coronaviruses (e.g., COVID-19, SARS-CoV, and MERS-CoV) [15]. Despite these promising results, such a potent drug candidate has not entered clinical application owing to its high toxicity to mammalian cells [16]. Thus, bafilomycin A_1_ has become an attractive target for medicinal chemists to synthesize unnatural bafilomycin derivatives with lower toxicity [17]. For the purposes of new drug development and diverse bioactivity assays, the demands for bafilomycin A_1_ are fast growing, which has naturally led to the demand for bafilomycin A_1_ high-producing strains since total synthesis of bafilomycin A_1_ remains highly challenging [18,19].

**Figure 2:**
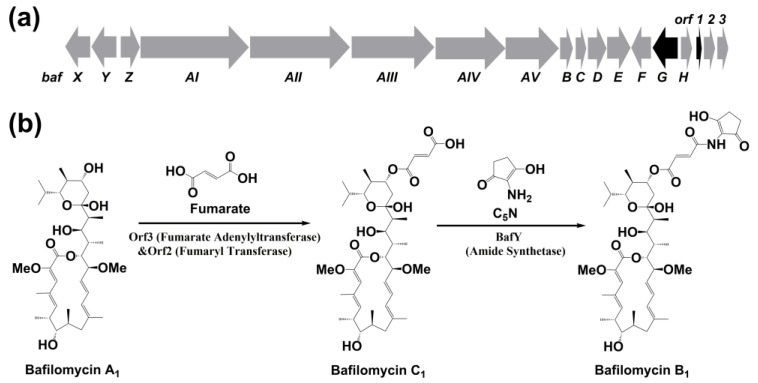
(**a**) The bafilomycin biosynthetic gene cluster of *S. lohii*. (*bafAI-AV*: PKS genes; *bafH*: thioesterase gene; *bafX-Z* and *orf* 2–3: post-PKS tailoring genes; *bafG* and *orf1*: regulatory genes; *bafB-bafF*: the genes for methoxymalonyl-CoA biosynthesis); (**b**) The bafilomycin post-PKS tailoring steps.

To engineer a bafilomycin A_1_ high-producer, knowledge on its biosynthetic mechanisms is required. To date, at least six bafilomycin biosynthetic gene clusters from *Streptomyces* and *Kitasatospora* species have been reported by this and other laboratories [8,10,20,21,22,23]. The modular and domain organization in five type I polyketide synthase (PKS) genes (exemplified by *bafAI*–*bafAV*, Figure 2a) collinearly matches the structure of bafilomycin A_1_. The five open reading frames *bafB*–*F* are responsible for biosynthesis of the methoxymalonate extender unit based on bioinformatics analysis. Recently, we completely elucidated the post-PKS tailoring steps (Figure 2b) of the bafilomycin biosynthetic pathway in *Streptomyces lohii* ATCC BAA-1276. Specifically, the adenylyltransferase Orf3 activates fumarate to fumaryl-AMP, whose fumaryl moiety is then transferred to C21-hydoxyl group of bafilomycin A_1_ by the fumaryl transferase Orf2, giving rise to bafilomycin C_1_. Next, the ATP-dependent amino synthetase BafY catalyzes the C–N bond formation between bafilomycin C_1_ and 2-amino-3-hydroxycyclopent-2-enone (C_5_N) to form bafilomycin B_1_. The C_5_N unit is assembled by the acyl-CoA ligase BafX and the bifunctional enzyme BafZ [9,20].

Except for those characterized as biosynthetic enzymes, *bafG* and *orf1* are the only two rest genes in the *baf* gene cluster with unclear functionality. According to the previous bioinformatics analysis [20], *bafG* and *orf1* likely encode an AfsR family transcriptional regulator and a LuxR family transcriptional regulator, respectively. To construct bafilomycin high-producers, however, it is necessary to understand the regulatory roles of *bafG* and *orf1* in the production of bafilomycins. In this work, we first optimized the fermentation medium for bafilomycin production. Subsequently, the regulatory roles of *orf1* and *bafG* in bafilomycin biosynthesis were characterized through gene inactivation and overexpression. By knocking out *orf2* and *orf3* that are responsible for the conversion of bafilomycin A_1_ to C_1_, together with *orf1* overexpression, a bafilomycin A_1_ high-producing strain (SLO-08) with the titer of 535.1 ± 25.0 mg/L in shaking flasks was successfully engineered. We anticipate that this strain will be utilized in the future to produce bafilomycin A_1_ in a cost-effective and eco-friendly manner for pharmacological researches and new drug development efforts.

## 2. Results

### 2.1. Optimization of the Fermentation Medium

**Figure 3:**
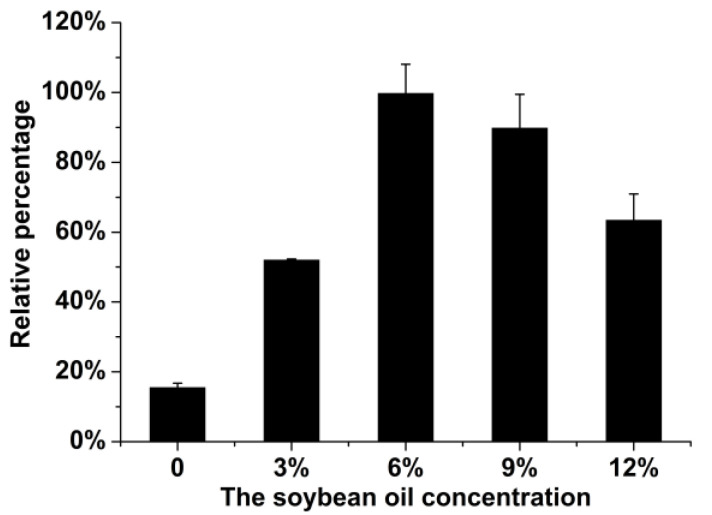
The relative percentage of bafilomycin production by wild-type *S. lohii* when supplied with different concentrations (*w*/*v*) of soybean oil in fermentation media. Note: The average bafilomycin production in the fermentation medium with 6% soybean oil is assigned as 100%.

We elected to optimize the fermentation medium since the accumulative production of bafilomycins A_1_, B_1_, and C_1_ by the wild type *S. lohii* strain (SLO-01, Table 1) was low (<40 mg/L) upon a 7-day fermentation. According to the previous study [24], soybean oil was considered as a cheap carbon source for *Streptomyces* to efficiently generate acyl-CoAs, which are common precursors of polyketide natural products. To test if soybean oil can also boost the production of bafilomycins, the fermentation media with different concentrations (*w*/*v*) of soybean oil (0, 3%, 6%, 9%, and 12%) were used to culture the wild type *S. lohii* strain. As shown in Figure 3, the bafilomycin production was significantly increased by addition of soybean oil. Specifically, 6% soybean oil resulted in the highest production of bafilomycins, corresponding to a 5.3-fold enhancement in bafilomycins production by *S. lohii* when compared to that in the same fermentation medium without soybean oil. Thus, the 6% soybean oil containing broth was used as the optimized fermentation medium for the production of bafilomycins in the following experiments.

### 2.2. Bioinformatics Analysis of the Regulatory Genes bafG and orf1

**Figure 4:**
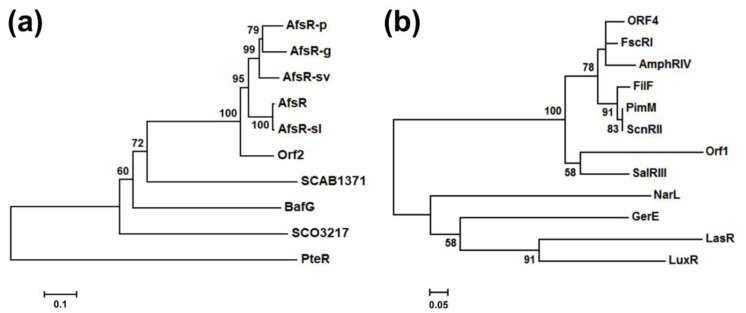
The neighbor-joining trees of (**a**) BafG with other identified *Streptomyces* AfsR homologues and (**b**) Orf1 with other identified LuxR family proteins. AfsR family proteins: AfsR (993 aa, GenBank accession number: BAA14186.1) and SCO3217 (638 aa, NP_627431.1) from *Streptomyces coelicolor* A3(2), AfsR-sl from *Streptomyces lividans* TK 24 (993 aa, EFD67634.1), AfsR-sv from *Streptomyces venezuelae* ATCC 15439 (1056 aa, ABR08660.1), SCAB1371 from *Streptomyces scabies* 87.22 (641 aa, CBG67361.1), AfsR-p from *Streptomyces peucetius* ATCC 27952 (982 aa, CAH10136.1), PteR from *Streptomyces avermitilis* MA-4680 (1096 aa, NP_821585.1), AfsR-g from *Streptomyces griseus* (974 aa, BAA83790.1), and Orf2 from *Streptomyces acidiscabies* ATCC 49003 (990 aa, BAO31545.1); LuxR family proteins: LuxR from *Vibrio fischeri* ATCC 7744 (250 aa, CAA68561.1), SalRIII from *Streptomyces albus* (231 aa, ABG02265.1), ORF4 from *Streptomyces noursei* ATCC 11455 (210 aa, AAF71781.1), FscRI from *Streptomyces* sp. FR-008 (222 aa, AAQ82551.1), PimM from *Streptomyces natalensis* (192 aa, CAM35468.1), ScnRII from *Streptomyces chattanoogensis* (192 aa, ADX66474.1), AmphRIV from *Streptomyces nodosus* (243 aa, WP_079161981.1), FilF from *Streptomyces filipinensis* (192 aa, AKX77828.1), LasR from *Pseudomonas aeruginosa* (239 aa, BAA06489.1), GerE from *Bacillus subtilis* (74 aa, CAA11701.1), and NarL from *Escherichia coli* K-12 (216 aa, AKK17394.1). The bars show the distance representing 0.1 (**a**) and 0.05 (**b**) substitutions per amino acid position and the bootstrap percentages under 50% from 1000 replicates are hidden.

The proteins encoded by *bafG* (BafG, 609 amino acids) and *orf1* (Orf1, 117 amino acids) show high sequence similarity with the AfsR family regulatory proteins and the LuxR family regulatory proteins from different *Streptomyces* spp., respectively (Figures S1 and S2). To investigate the evolutionary relationship between BafG and the select number of AfsR family regulators from *Streptomyces*, the phylogenetic tree was built based on their amino acid sequences using the neighbor-joining method [30] (Figure 4a). This phylogenetic analysis confirmed that BafG is indeed an AfsR family member (Figure 4a). According to multiple sequence alignment (Figure S1) and BLAST analysis, BafG exhibits several typical conservative DNA binding domains including the transcriptional regulatory protein, *C*-terminal domain (trans_reg_C domain: 6–73 aa), the bacterial transcriptional activation domain (BTA domain: 81–225 aa), and the nucleotide-binding adaptor shared by APAF-1, R proteins, and CED-4 domain (NB-ARC domain: 302–479 aa); three 34-aa tetratricopeptide repeat motifs (TPR motifs: 81–114 aa, 136–169 aa, and 173–206 aa), which is responsible for recruiting RNA polymerase to bind to the promoter of target genes [31]; and several key residues related to the nucleotides recognition: ^24^SerVal^25^, Thr44, Thr47, ^55^SerLeu^56^, Thr66, and Gly70. Compared with the 993-aa AfsR, the *C*-terminus of 609-aa BafG lacks four TPR repeats which has been validated to be dispensable for the basic function of AfsR as a transcriptional activator [31]. Furthermore, there is a unique “TTA” (Leu173) in the coding sequence of *bafG*, which is the rarest codon in the high-GC-content *Streptomyces* genomes (Figure S3) [32,33]. This rare codon strongly suggests the possibility that *bafG* might participate in the regulation of bafilomycin biosynthesis since the involvement of the genes with a “TTA” codon in regulating cell differentiation and antibiotics production has been proposed for other *Streptomyces* species [32].

With respect to Orf1, this small protein displays >45%/68% identity/similarity to several identified LuxR family members from *Streptomyces* (Figure S2). A phylogenetic tree was built using a number of LuxR family proteins including LuxR from *Vibrio fischeri* ATCC 7744 [34], GerE from *Bacillus subtilis* [35], LasR from *Pseudomonas aeruginosa* [36], NarL from *Escherichia coli* K-12 [37], and so on (Figure 4b). The phylogenetic analysis clearly indicated Orf1 is a LuxR family transcriptional regulator. Specifically, the protein sequence alignment result showed that there is a highly conserved helix-turn-helix (HTH) motif at the *C*-terminus of Orf1 (41–95 aa), which is a common feature of LuxR family regulators (Figure S2) [38]. At the *N*-termini of many LuxR family members, there generally exist a receptor for inducer binding [39], such as the Per-Arnt-Sim (PAS) domains of SalRIII [40], PimM [41], and FscRI [42]. However, Orf1 only has a *C*-terminal HTH motif as GerE, suggesting that Orf1 might be an inducer-independent LuxR homologue capable of directly activating the transcription of related genes as the *N*-truncated LuxR (∆2–162) [43].

### 2.3. The Regulatory Roles of bafG and orf1 in Bafilomycin Production

**Figure 5:**
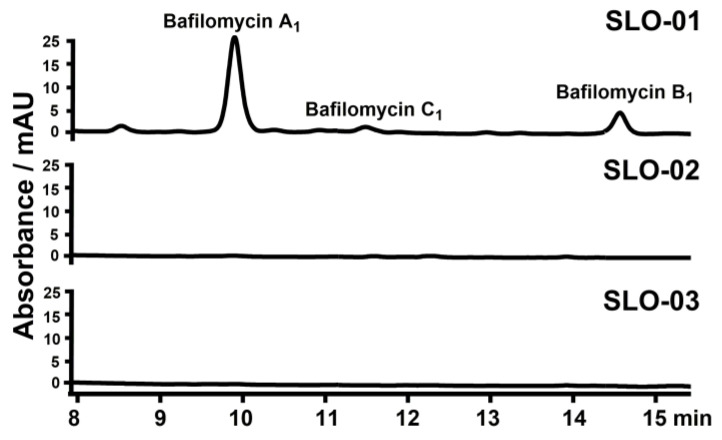
HPLC analysis (254 nm) of the fermentation broth of wild-type *S. lohii* and the *bafG*/*orf1* inactivation strains.

**Figure 6:**
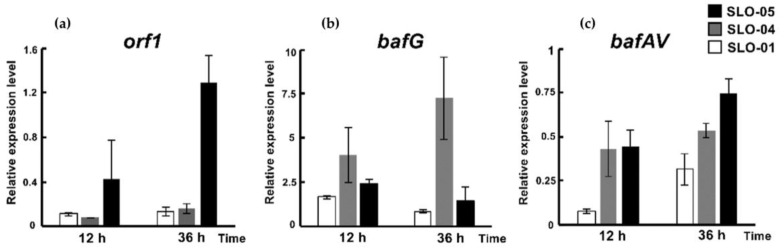
The relative transcriptional levels of (**a**) *orf1*, (**b**) *bafG,* and (**c**) *bafAV* in wild-type and the *orf1*/*bafG* overexpressed *S. lohii* strains at 12 h and 36 h.

**Figure 7:**
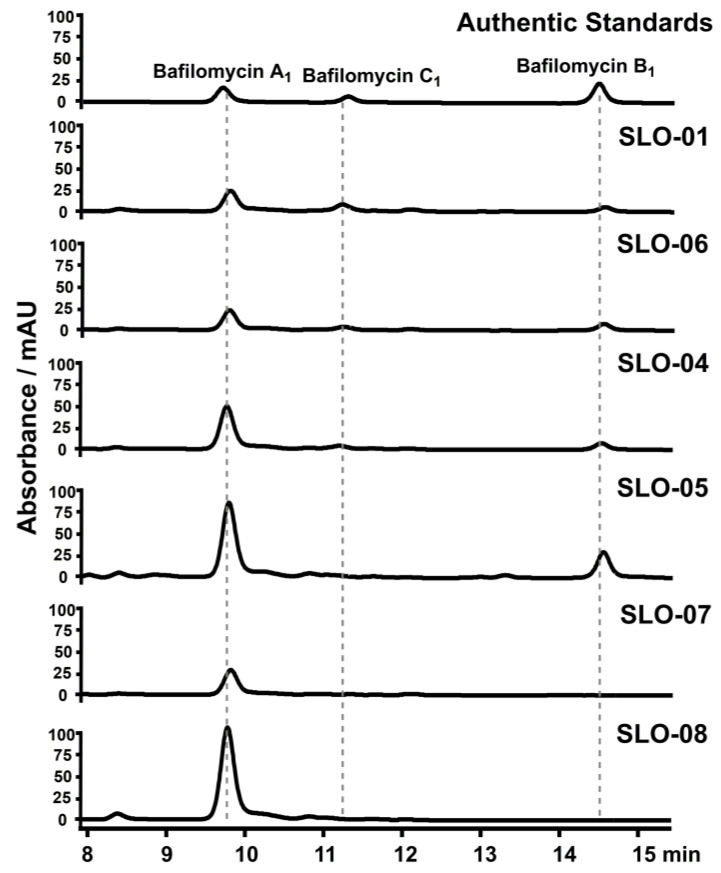
HPLC analysis (254 nm) of the bafilomycin production by wild-type and mutant *S. lohii* strains.

**Figure 8:**
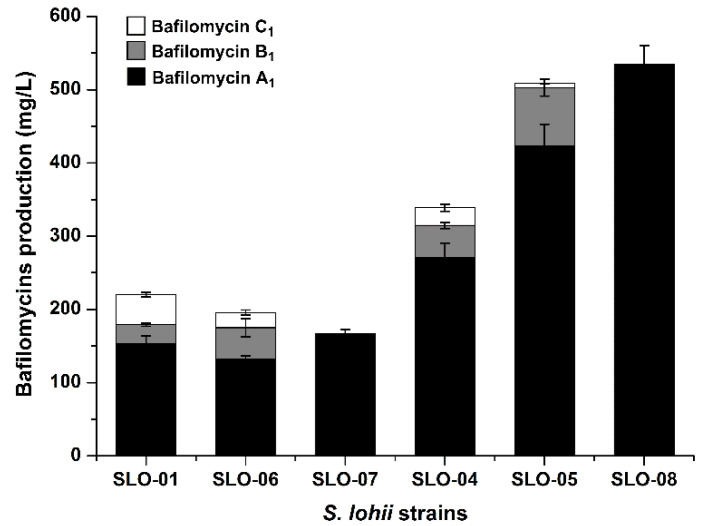
The bafilomycin production (mg/L) by wild-type and mutant *S. lohii* strains.

To probe the functions of the *afsR* family member *bafG* and the *luxR* family regulatory gene *orf1* for bafilomycin production in *S. lohii*, a 682-bp internal fragment of *bafG* or a 335-bp internal fragment of *orf1* was in-frame replaced by the *aac(IV)* cassette via homologous recombination, giving rise to the *bafG* deletion mutant SLO-02 and the *orf1* deletion mutant SLO-03, respectively (Figure S4). As results, SLO-02 and SLO-03 without any cell growth and morphological difference completely lost the ability to produce any bafilomycins (Figure 5). These results suggested that the disruption of *bafG* or *orf1* might transcriptionally inactivate/repress some key biosynthetic genes in the *baf* cluster. Next, *bafG* and *orf1* were overexpressed in *S. lohii* by generating the strains SLO-04 (*S. lohii*/pSET152-ermE*-*bafG*, Table 1) and SLO-05 (*S. lohii*/pSET152-ermE*-*orf1*, Table 1). Compared with the wild type *S. lohii*, the transcriptional levels of *orf1* and *bafG* in these two mutants, without any cell growth difference observed, were significantly increased at 12 h and 36 h under the strong promoter ermE* (Figure 6). Furthermore, wild-type *S. lohii* produced 220.3 ± 10.9 mg/L of bafilomycins in the optimized fermentation medium within 7 days in the 250 mL shaking flasks (Figure 7 and Figure 8), while the *bafG* overexpression strain SLO-04 and the *orf1* overexpression strain SLO-05 generated 338.9 ± 10.7 mg/L and 508.5 ± 41.2 mg/L of bafilomycins in parallel fermentations, respectively, corresponding to 1.5- and 2.3-fold higher total bafilomycins titers than the wild type strain (Figure 7 and Figure 8). The bafilomycin A_1_ titers of SLO-04 (271.1 ± 19.7 mg/L) and SLO-05 (423.5 ± 29.5 mg/L) increased 0.8- and 1.8-fold relative to that of wild-type *S. lohii* (152.9 ± 11.4 mg/L), respectively (Figure 7 and Figure 8). These titer improvements could be qualitatively rationalized by the quantitative real-time PCR (qRT-PCR) results (Figure 6): the overexpression of *orf1* in SLO-05 led to a 4.9- and 1.4-fold improvement of the transcriptional level of *bafAV*, the final PKS gene for bafilomycin biosynthesis, when compared with wild-type *S. lohii* at 12 h and 36 h, respectively; and the transcriptional level of *bafAV* in SLO-04 was increased by 4.7- and 0.7-fold compared to that of wild-type *S. lohii* at 12 h and 36 h, respectively. These results strongly suggested that *bafG* and *orf1* are indeed positive transcriptional factors for bafilomycin biosynthesis in *S. lohii*.

### 2.4. Construction of Bafilomycin A_1_ High-Producing Strains

In our previous study [9], SLO-07 (*S. lohii* ∆*orf2&orf3*, Table 1), in which the region encoding *orf2* and *orf3* was replaced by *aac(IV)* to disrupt the bafilomycin post-PKS tailoring steps in *S. lohii*, was constructed. In this study, this strain solely produced bafilomycin A_1_ (167.3 ± 5.4 mg/mL) under the optimized fermentation conditions (Figure 7 and Figure 8). To further construct a bafilomycin A_1_ high-producing strain, *orf1*, whose overexpression led to higher bafilomycin A_1_ production than that of *bafG*, was chosen to be introduced into SLO-07, giving rise to SLO-08 (Table 1). Upon overexpression of *orf1* in the strain that purely produces bafilomycin A_1_, the resultant yield of bafilomycin A_1_ reached 535.1 ± 25.0 mg/L (Figure 7 and Figure 8), representing the highest reported bafilomycin A_1_ production to date.

## 3. Discussion

Generally, oil is one of excellent carbon sources commonly used in the fermentation media of *Streptomyces* to support cell growth and metabolites [24]. More importantly, soybean oil has been used as a low-cost feedstock to enhance the supply of biosynthetic precursors for improvement of polyketide production in *Streptomyces* since fatty acids can be directly bioconverted into acyl-CoAs as the precursors of polyketides [24,44,45]. For example, the FK506 production in *Streptomyces tsukubaensis* was increased 0.9-fold by feeding soybean oil into the production medium [24]. Here, the initial fermentation medium for *S. lohii* was optimized through investigating the relationship between soybean oil concentrations and bafilomycin production; and 6% soybean oil showed the best improving effect. Based on our analysis of the draft genome of *S. lohii*, at least forty esterase/lipase genes were revealed, suggesting that soybean oil could be efficiently utilized via primary metabolism for both cell growth (the biomass of *S. lohii* was significantly enhanced in the optimized medium) and bafilomycin production. In the optimized fermentation medium, the dominant product of the wild type *S. lohii* was bafilomycin A_1_, accounting for about 70% of total production of bafilomycins, although bafilomycin B_1_ is the primary product when using the MD2 medium for *S. lohii* fermentation in our previous study [20]. Thus, using different fermentation materials to regulate the bafilomycin production and product distribution will be our next goal.

In this work, BafG and Orf1 were identified as the positive AfsR family regulator and the positive LuxR family regulator, respectively. AfsR was firstly characterized by Horinouchi et al. as a global activator involved in the regulatory cascades and antibiotics production in the type strain *Streptomyces coelicolor* A3(2) [46,47]. The activity of AfsR for enhancing the transcription of *afsS* can be significantly improved after the phosphorylation of its threonine and serine residues by AfsK [47]. Generally, AfsR family proteins work as transcriptional activators in secondary metabolite biosynthesis. For example, the overexpression of *afsR-sv* in *S. venezuelae* ATCC 15439 improved the production of pikromycin [48]; SCAB1371 was identified as a positive transcriptional regulator for pyochelin biosynthesis in the plant pathogen *Streptomyces scabies* 87–22 [49]; the overexpression of *afsR* in *Streptomyces lomondensis* led to up-regulation of two genes related to lomofungin biosynthesis and increased the lomofungin production by 2.5-fold [50]. Similarly, the disruption of the *bafG* homologue *bfmH* in *Kitasatospora setae* KM-6054 also led to the abolishment of bafilomycins [8]. The characterization of *bafG* will expand the pool of the *afsR* family regulatory genes and it may be overexpressed in other *Streptomyces* species for isolation of novel compounds through activating silent genes or for improvement of the target antibiotics production, which is currently ongoing in our laboratory. However, there is no any *afsK* homologue in the *baf* cluster, which suggests that BafG might be phosphorylated by an AfsK homologue outside the gene cluster.

LuxR was firstly characterized in the *lux* operon of *Vibrio fischeri* ATCC 7744, which is a cell density-dependent transcriptional activator involved in luciferase biosynthesis and play important roles in acyl-homoserine lactones-mediated quorum sensing [34,39]. To date, hundreds of LuxR family members have been discovered by genome mining and bioinformatics predictions. Of note, dozens of LuxR family regulatory factors have been proved to participate in *Streptomyces* secondary metabolite biosynthesis, such as PikD, which was identified as a pathway-specific positive regulator for pikromycin biosynthesis in *Streptomyces venezuelae* [51]; TmcN, the activator of tautomycetin biosynthesis in *Streptomyces* sp. CK4412 [52]; and RapH, a putative transcriptional regulator for rapamycin biosynthesis in *Streptomyces hygroscopicus* [53]. Generally, the *N*-terminal motifs of LuxR family proteins are bound by the quorum-sensing molecules to relieve the repression by the *C*-terminal motif responsible for activating the transcription of related genes [39]. In this study, the results of *orf1* inactivation and overexpression strongly suggest its positive regulatory role in bafilomycin biosynthesis of *S. lohii*. To our surprise, Orf1 was identified as a LuxR family protein with only a conservative *C*-terminal HTH motif, suggesting it may function as an inducer-independent activator, similar to GerE and the *N*-truncated LuxR (∆2–162) [35,43].

In previous studies, the gamma-butyrolactone synthetase/autoregulator receptor homologues were found to play vital roles in bafilomycin production. Specifically, the deletion of the gamma-butyrolactone synthetase gene homologue *stcA* from *Streptomyces* sp. SBI034 led to the complete abolishment of bafilomycin production as well as aerial mycelium formation and sporulation [54]; the deletion of gamma-butyrolactone autoregulator receptor genes *ksbA* and *ksbC* in *Kitasatospora setae* indicated that KsbA and KsbC respectively control bafilomycin production and aerial mycelium formation negatively and positively [55,56]. However, the regulatory factors involved in bafilomycin biosynthesis has not caught much attention in *Streptomyces* species. Our study provides some initial understandings of the regulatory roles of the *afsR* and *luxR* family genes in governing the biosynthesis of bafilomycins, which also provide an effective strategy for engineering high bafilomycin producers. Since total synthesis of bafilomycin A_1_ has been proved to be complex and low-yield [18,19], the construction of bafilomycin A_1_ high-producing strains holds great potential of application.

## 4. Materials and Methods

### 4.1. Materials

The chemicals and antibiotics in this study were purchased from Solarbio (Beijing, China) and Sinopharm Chemical Reagent (Beijing, China) unless otherwise specified. Apramycin sulfate was bought from Sangon (Shanghai, China). T4 DNA ligase and all fast-digest restriction endonucleases were bought from Thermo Fisher Scientific (Waltham, MA, USA). I-5™ 2 × High-Fidelity Master Mix obtained from TsingKe (Beijing, China) was used for PCR amplification. ClonExpress Ultra One Step Cloning Kit was purchased from Vazyme (Nanjing, China). MonPure™ Gel & PCR Clean Kit and Plasmid Miniprep Kit were bought from Baisai Biotechnology (Qingdao, China). GelRed for agarose gel electrophoresis was purchased from the MDBio (Xinbei, China). The MiniBEST Universal RNA Extraction Kit for RNA extraction and genomic DNA digestion, the PrimeScript™RT reagent for cDNA preparation and the TB Green^®^Premix Ex Taq™II (Tli RNaseH Plus) for qRT-PCR were purchased from Takara (Dalian, China).

### 4.2. Strains, Plasmids, and Bacterial Growth Conditions

Strains and plasmids used in this study are listed in Table 1. *E. coli* DH5a [25] was used as the host strain for plasmid construction, replication, and preservation. *E. coli* ET12567/pUZ8002 [26] was employed for interspecies conjugation between *E. coli* and *S. lohii*. All *E. coli* strains were cultivated in Luria–Bertani medium (10 g tryptone, 5 g yeast extract, and 10 g NaCl per liter) at 37 °C. The wild type and mutant *S. lohii* strains were grown on MS agar (mannitol 20 g, soybean flour 20 g, and agar 20 g per liter) at 28 °C for sporulation and conjugation. 2 × YT liquid medium (16 g tryptone, 10 g yeast extract, and 5 g NaCl per liter) was used to grow *S. lohii* cells for genomic DNA (gDNA) preparation. The initial fermentation medium (pH = 7.1) contained 20 g glucose, 20 g soybean flour, 2 g NZ-amine, 1.5 g corn syrup, 1 g yeast extract, 8 g NaNO_3_, 8 g CaCO_3_, 6 g (NH_4_)_2_SO_4_, 5 g NaCl, and 0.3 g K_2_HPO_4_ per liter. The optimized fermentation medium was the initial fermentation medium supplemented with 6% soybean oil. The concentrations of antibiotics used in this study were as follows: apramycin (50 μg/mL), spectinomycin (100 μg/mL), kanamycin (50 μg/mL), chloramphenicol (25 μg/mL), and nalidixic acid (25 μg/mL).

### 4.3. DNA Sequencing and Bioinformatics Analysis

DNA sequencing and primer synthesis were performed by TsingKe (Qingdao, China). Gene annotation and the collection of amino acid sequences of AfsR and LuxR family members were carried out using NCBI databases (http://www.ncbi.nlm.nih.gov/). The gene promoters were predicted using Softberry online tools (http://linux1.softberry.com/). The phylogenetic analysis was performed by MEGA version 7.0 (Philadelphia, PA, USA) [57] using the neighbor-joining method [30]. DNAman 7.0 (San Ramon, Cal, USA) was used for protein sequence alignments.

### 4.4. Construction of the Suicide Knockout Vectors

The primers for vector construction are listed in Table S1. The suicide vector pCIMt002 was kindly provided by Prof. Yihua Chen at Institute of Microbiology, Chinese Academy of Sciences [29]. To generate the suicide knockout vectors for *bafG* and *orf1* inactivation, the upstream and downstream homologous fragments (approximately 2.0 kb) of *bafG* were amplified from the *S. lohii* gDNA using the primer pairs of *bafG*-LA-FP/*bafG*-LA-RP and *bafG*-RA-FP/*bafG-*RA-RP, respectively; and the upstream and downstream homologous fragments (about 2.0 kb) of *orf1* were amplified from the *S. lohii* gDNA using the primer pairs of *orf1*-LA-FP/*orf1*-LA-RP and *orf1*-RA-FP/*orf1*-RA-RP, respectively. Subsequently, the homologous fragments were cloned into the *Nco*I and *Nhe*I restriction sites of pCIMt002 to generate pCIMt002-*∆bafG* and pCIMt002-*∆orf1* using the ClonExpress Ultra One Step Cloning Kit.

### 4.5. Gene Inactivation in S. lohii

The gene *orf1* or *bafG* in *S. lohii* was replaced by the apramycin resistance cassette (*aac(IV)*) following the blue-white screening strategy developed by Chen et al. [29]. The suicide vectors pCIMt002-∆*bafG* or pCIMt002-∆*orf1* were transferred into *S. lohii* via *E. coli*–*Streptomyces* conjugation [26]. Upon an incubation at 28 °C for 12 h, each MS agar plate (containing 50 mM CaCl_2_ and 50 mM MgCl_2_) was overlaid with 1 mL sterilized water containing 1.25 mg apramycin and 0.5 mg nalidixic acid. After a further 28 °C incubation for 3–5 days, the white colonies indicative of the desired double-crossover recombinants were picked up from the blue colonies indicative of the undesired single-crossover mutants. The genotypes of the two picked mutants (SLO-02 for Δ*bafG* and SLO-03 for Δ*orf1*, Table 1) were confirmed by PCR (Figure S4).

### 4.6. Construction of Integrative Plasmids for Regulatory Gene Overexpression

The coding sequences of *bafG* (1830 bp) and *orf1* (354 bp) were amplified using the *S. lohii* gDNA as template. For *bafG*, the primer pair was BafG-BamHI-FP/BafG-KpnI-RP; and for *orf1*, the primer pair was Orf1-BamHI-FP/Orf1-KpnI-RP (Table S1). The *bafG* and *orf1* fragments were inserted into the *Bam*HI restriction site of pSET152-ermE* (Bierman et al., 1992) to generate the regulatory gene overexpression vectors pSET152-ermE*-*bafG* and pSET152-ermE*-*orf1*, respectively. Since the apramycin resistance gene has already been integrated into the genome of *S. lohii* Δ*orf2&orf3*, the spectinomycin resistance gene (*aadA*) fragment was PCR amplified from pIJ778 (Gust et al., 2003) using the primers Spec-NdeI-FP/Spec-SacI-RP as the second selection marker. Next, the *aadA* cassette was in-fusion cloned into the *Sac*I-pre-digested pSET152-ermE*, yielding the integrative vector pSET152s-ermE*. The coding sequence of *orf1* was cloned into pSET152s-ermE* to afford pSET152s-ermE*-*orf1* for gene overexpression in *S. lohii* Δ*orf2&orf3*.

### 4.7. Overexpression of Regulatory Genes

For gene overexpression of *bafG* or *orf1*, the integrative plasmid pSET152-ermE*-*bafG* or pSET152-ermE*-*orf1* was introduced into wild-type *S. lohii*, respectively, by interspecies conjugation from *E. coli* ET12567/pUZ8002 [26]. Upon an incubation at 28 °C for 12 h, each plate was overlaid with 1 mL sterilized water containing 1.25 mg apramycin and 0.5 mg nalidixic acid. After additional 3–5 days, the recombinants were inoculated onto MS plates with 25 μg/mL nalidixic acid and 50 μg/mL apramycin. The resultant two apramycin resistant strains SLO-04 (*S. lohii*/pSET152-ermE*-*bafG*, Table 1) and SLO-05 (*S. lohii*/pSET152-ermE*-*orf1*, Table 1) were PCR confirmed using their gDNA as template.

### 4.8. Genotypic Confirmation of S. lohii Mutants

The primers for PCR confirmation of *S. lohii* mutants are listed in Table S2. The primers *bafG*-KO-FP/*bafG*-KO-RP were used for screening the ∆*bafG* mutants. The expected length of the PCR fragments from the wild type and the ∆*bafG* mutants is 916 bp and 1227 bp, respectively (Figure S4a,b). The primers *orf1*-KO-FP/*orf1*-KO-RP were used for screening the ∆*orf1* mutants. The expected length of the PCR fragments from the wild type and the ∆*orf1* mutants is 562 bp and 1220 bp, respectively (Figure S4c,d). The primers M13F-47/M13R-48 were used to screen the *orf1* and *bafG* overexpression strains (Figure S5): the expected length of the PCR product from SLO-06 (Table 1) is 471 bp; the expected length of PCR products of SLO-05 and SLO-08 (Table 1) is 831 bp; and the expected length of PCR products of SLO-04 is 2307 bp. All the PCR fragments were gel purified and further confirmed by DNA sequencing.

### 4.9. Fermentation and HPLC Analysis

A single colony of the wild type or each mutant of *S. lohii* was used to inoculate 30 mL 2 × YT medium, and cultured at 220 rpm, 28 °C. After 2 days, 3 mL seed culture was inoculated into 30 mL fermentation medium and cultivated at 28 °C, 250 rpm for another 7 days. Next, 200 μL fermentation culture was extracted by adding 600 μL methanol, vortexed for 30 min, and centrifuged at 14,000× *g* for 10 min. The supernatants were directly used for reverse phase HPLC analysis (254 nm) with a Thermo C-18 column (4.6 × 150 mm) under a liner gradient of 60–100% acetonitrile over 15 min, 100% acetonitrile for 5 min, and 100–60% acetonitrile over 2 min in deionized H_2_O (with 0.1% trifluoroacetic acid) at a flow rate of 1 mL/min. The fermentation of the wild type and all mutant *S. lohii* strains were carried out in duplicate, and the production of bafilomycins was quantified based on the integrated peak areas using authentic bafilomycin A_1_, B_1_, and C_1_ as standards [9].

### 4.10. Transcriptional Analysis of the Wild Type and Mutant S. lohii Strains by qRT-PCR

The mycelia of the wild type or mutant *S. lohii* strains in the optimized fermentation media were collected at 12 and 36 h. The total RNA was extracted with the genomic DNA removed using the MiniBEST Universal RNA Extraction Kit, and was reversely transcribed using random primer mix by following the product manual. The primers for qRT-PCR were designed by Primer3Plus online service (http://www.primer3plus.com/cgi-bin/dev/primer3plus.cgi) and listed in Table S3. The transcription of target genes was determined by qRT-PCR on a LightCycler 480 II (Roche Life Science, Basel, Switzerland) in triplicate. For determination of the relative transcription levels, the data were normalized to the housekeeping gene *hrdB* in *S. lohii* and quantified by the 2^−ΔΔCT^ method [58].

## Figures and Tables

**Table 1:** Bacterial strains and plasmids.

Strain or Plasmid	Characteristics	Reference
*Escherichia coli* strains		
DH5a	Cloning host	[25]
ET12567/pUZ8002	Interspecies conjugation	[26]
*Streptomyces* strains		
SLO-01	*Streptomyces lohii* ATCC BAA-1276 (wild-type strain)	[20]
SLO-02	*S. lohii* ∆*bafG*	This study
SLO-03	*S. lohii* ∆*orf1*	This study
SLO-04	*S. lohii*/pSET152-ermE*-*bafG*	This study
SLO-05	*S. lohii*/pSET152-ermE*-*orf1*	This study
SLO-06	*S. lohii*/pSET152-ermE*	This study
SLO-07	*S. lohii* ∆*orf2&orf3*	[9]
SLO-08	*S. lohii* ∆*orf2&orf3*/pSET152s-ermE*-orf1	This study
Plasmids		
pSET152-ermE*	Apramycin resistance	[27]
pSET152s-ermE*	Spectinomycin resistance	This study
pIJ778	Spectinomycin resistance	[28]
pCIMt002	Ampicillin and Apramycin resistance	[29]

## Data Availability

Data is contained within the article or supplementary materials.

## Supplementary Materials

The following are available online at https://www.mdpi.com/1660-3397/19/1/29/s1, Figure S1: Multiple protein sequence alignment of BafG with several AfsR family proteins, Figure S2: Multiple protein sequence alignment of Orf1 with several LuxR family proteins, Figure S3: The partial open reading frame of BafG (141–210 aa), Figure S4: Inactivation of *bafG* and *orf1*, Figure S5: The overexpression of *bafG*/*orf1*, Table S1: The primers for construction of knock-out and regulatory genes overexpression vectors, Table S2: The primers for construction of knock-out vectors and PCR confirmation of *S. lohii* mutants, Table S3: The primers for quantitative real-time PCR.
