# Supplementary material for: Engineering Bafilomycin High-Producers by Manipulating Regulatory and Biosynthetic Genes in the Marine Bacterium Streptomyces lohii
Source: Mar Drugs. 2021 Jan 11;19(1):29. doi: 10.3390/md19010029 (PMC7827423; doi:10.3390/md19010029)
Supplement: Supplementary file 1 [file marinedrugs-19-00029-s001.pdf]

Supplementary Materials

Engineering bafilomycin high-producers by manipulating regulatory and biosynthetic genes in the marine bacterium *Streptomyces lohii*

Zhong Li[1,2,3], Shuai Li[1], Lei Du[1], Xingwang Zhang[1], Yuanyuan Jiang[1,2,3], Wenhua Liu[1], Wei Zhang[1] and Shengying Li[1,4]*

[1]State Key Laboratory of Microbial Technology, Shandong University, Qingdao, Shandong 266237, China

[2]Shandong Provincial Key Laboratory of Synthetic Biology, CAS Key Laboratory of Biofuels at Qingdao Institute of Bioenergy and Bioprocess Technology, Chinese Academy of Sciences, Qingdao, Shandong 266101, China

[3]University of Chinese Academy of Sciences, Beijing 100049, China

[4]Laboratory for Marine Biology and Biotechnology, Qingdao National Laboratory for Marine Science and Technology, Qingdao, Shandong 266237, China

*To whom correspondence may be addressed. E-mail: lishengying@sdu.edu.cn (S.L.)

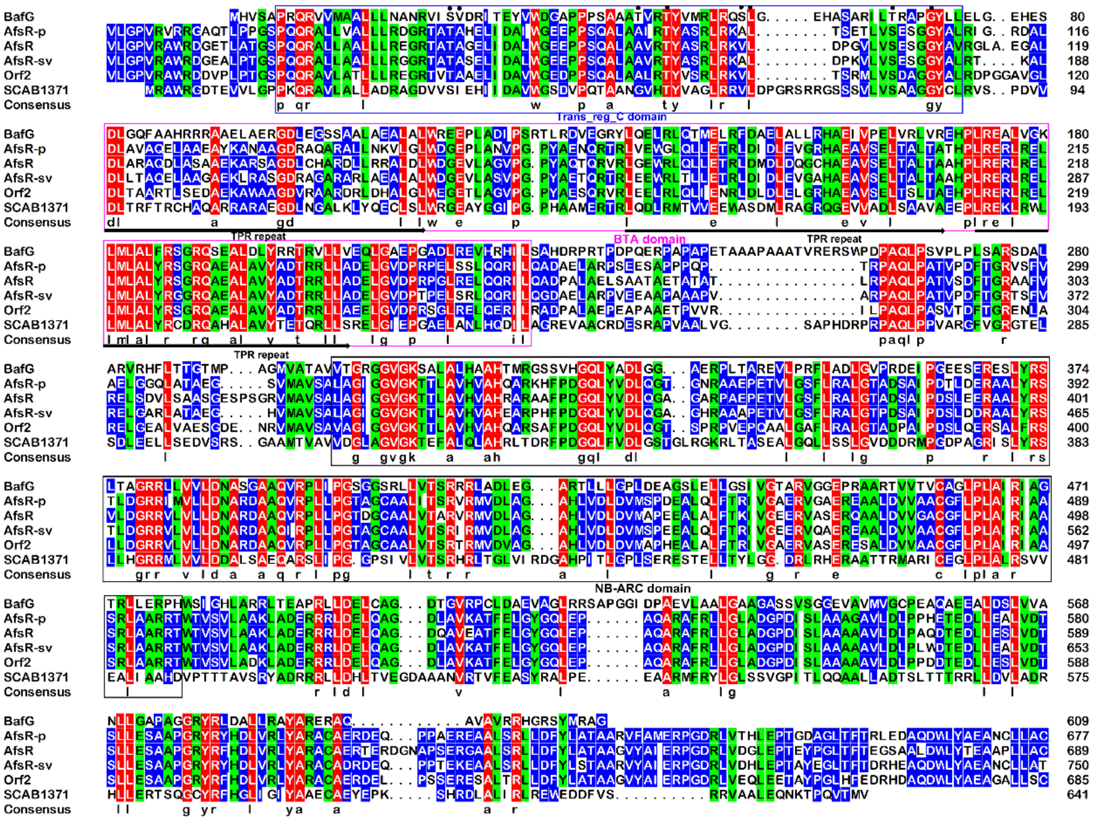

Figure. S1 Multiple protein sequence alignment of BafG with several AfsR family proteins. The trans_reg_C domain, BTA domain and NB-ARC domain are marked with blue, purple and black frames, respectively; three TRP repeats are emphasized by black arrows; the key residues of BafG related to the nucleotides recognition (^{24}SerVal^{25}, Thr44, Thr47, ^{55}SerLeu^{56}, Thr66 and Gly70) are marked with “●”. *Note*: The amino acids highlighted in red stand for 100% homology; the residues highlighted in green and blue stand for 75% < homology <100% and 50% < homology <75%, respectively.

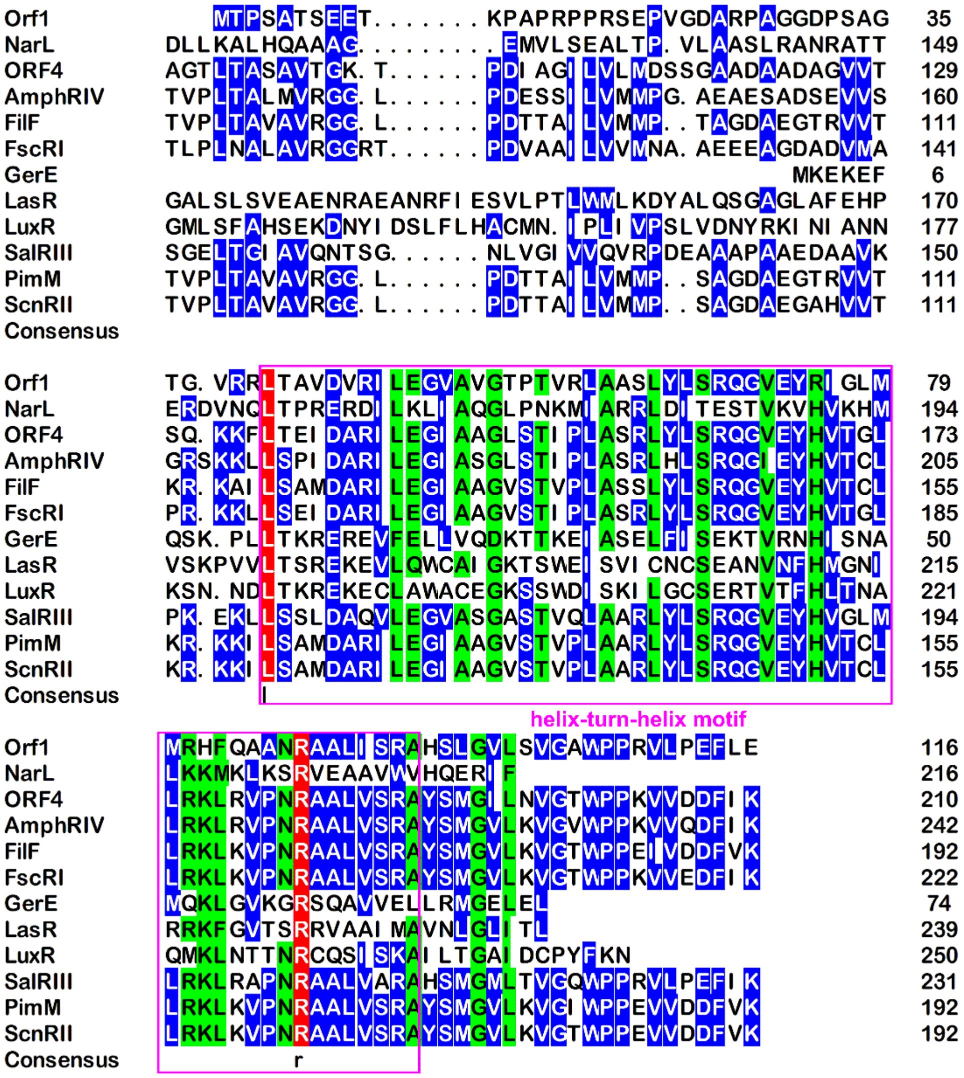

Figure. S2 Multiple protein sequence alignment of Orf1 with several LuxR family proteins. The helix-turn-helix motif is marked with purple frames. *Note*: The amino acids highlighted in red stand for 100% homology; the residues highlighted in green and blue stand for 75% < homology <100% and 50% < homology <75%, respectively.

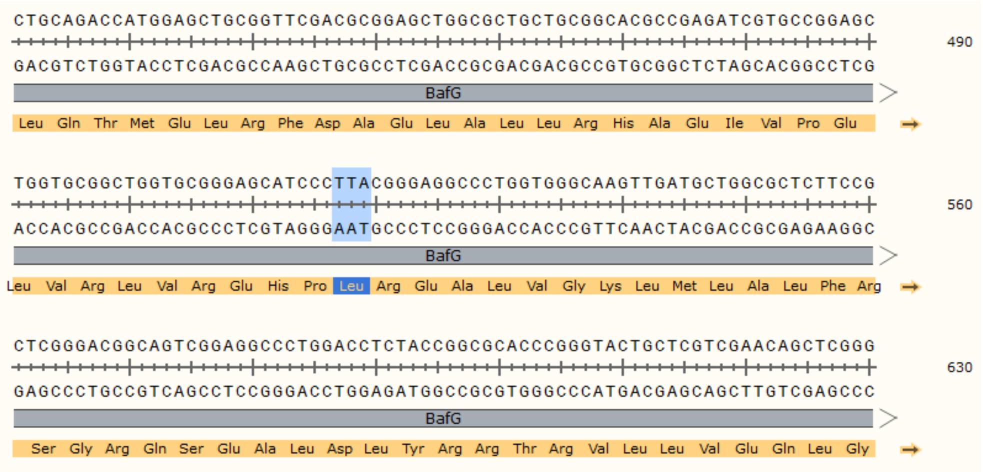

Figure. S3 The partial open reading frame of BafG (141-210 aa). The “Leu173” shaded in blue is a unique Leu coded by the rarest codon “TTA” in the *Streptomyces* genome.

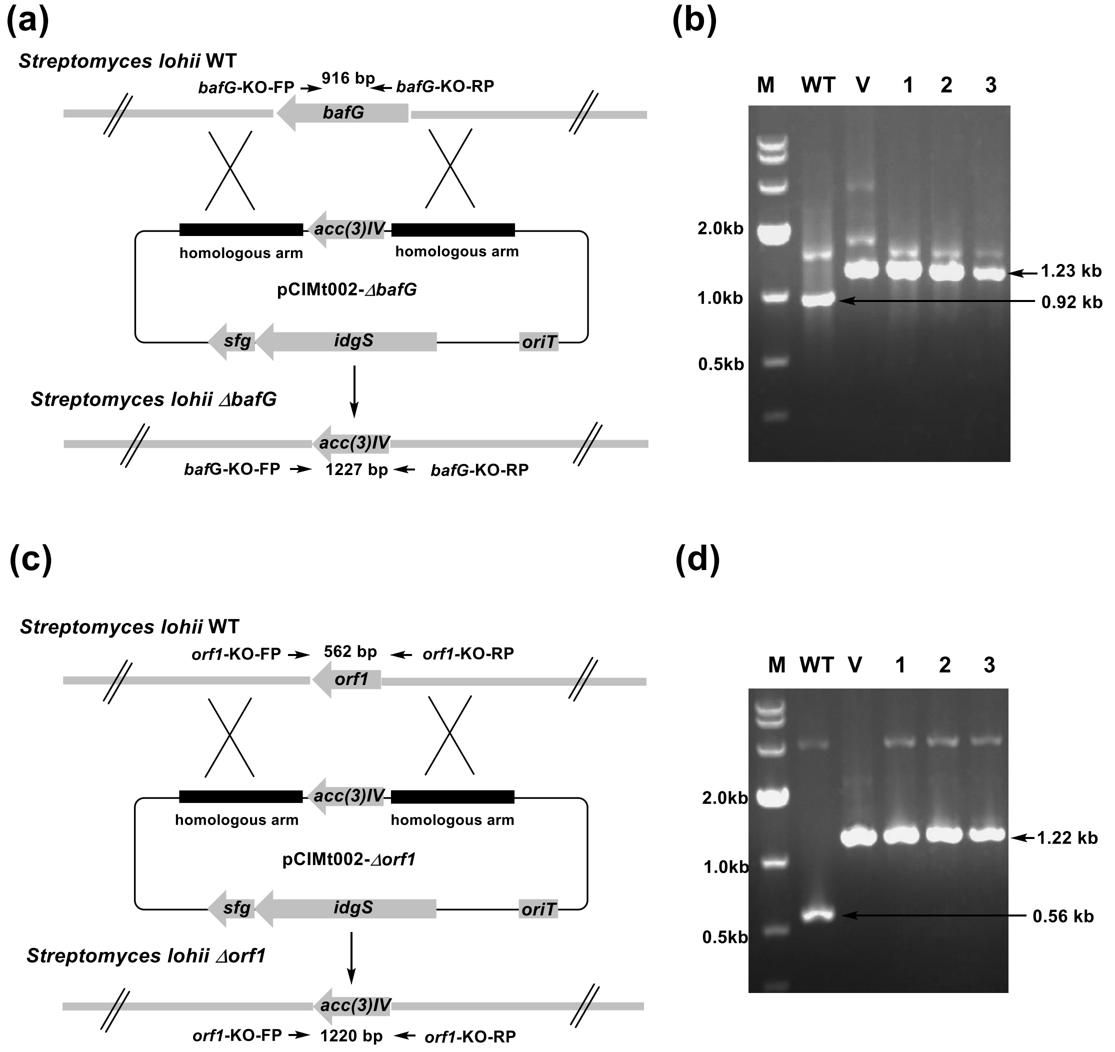

Figure. S4 Inactivation of *bafG* and *orf1*. (a) Construction of the *bafG* inactivation mutant *S. lohii* Δ*bafG* (SLO-02). (b) PCR confirmation of the Δ*bafG* mutants. M: DNA marker; WT: *S. lohii* wild type; V: pCIMt002-Δ*bafG*; 1-3: *bafG* inactivation mutants. (c) Construction of the *orf1* inactivation mutant *S. lohii* Δ*orf1* (SLO-03). (d) PCR confirmation of the Δ*orf1* mutants. M: DNA marker; WT: *S. lohii* wild type; V: pCIMt002-Δ*orf1*; 1-3: *orf1* inactivation mutants. *Note*：LA：left homologous arm; RA：right homologous arm.

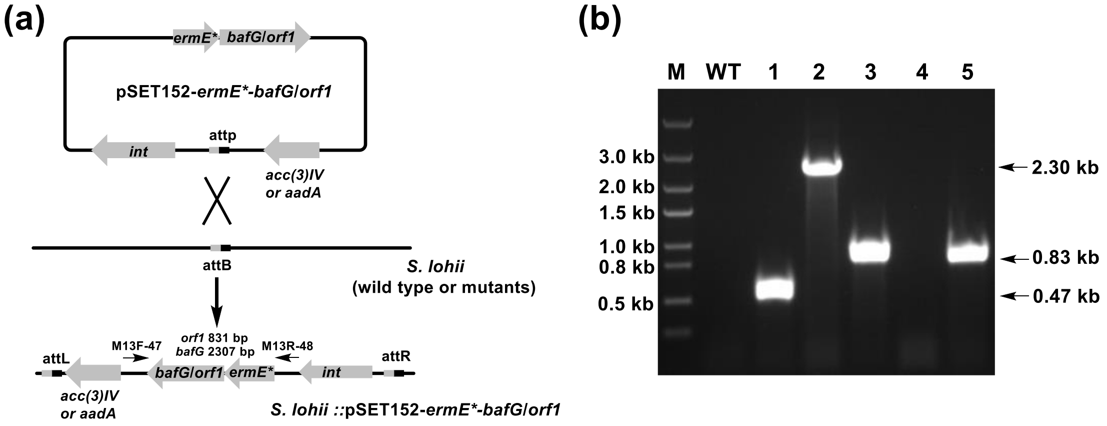

Figure. S5 The overexpression of *bafG/orf1*. (a) Construction of the *bafG* and *orf1* overexpression strains. (b) PCR confirmation of the *S. lohii* mutants. M: DNA marker; WT: *S. lohii* wild type (SLO-01); 1: SLO-06; 2: SLO-04; 3: SLO-05; 4: SLO-07; 5: SLO-08.

Table S1. The primers for construction of knock-out and regulatory genes overexpression vectors.

Primers	Sequence (5'-3')
***bafG*-LA-FP**	AAGAGCTTTTATAAAAGCTT*CCATGG*GGTGACCGGGCAGGTGAC
***bafG*-LA-RP**	AACGTGAGCCTAGGGCGTGC*CCATGG*GAGGGAAGCTGCGCAGGA
***bafG*-RA-FP**	TTGGCTGACAATTGACATCT*GCTAGC*CTGGACGAACTGTGCGCG
***bafG*-RA-RP**	GTGGATCCGCACCCAAGCTT*GCTAGC*GGCTCCAAGTCCGATGCG
***orf1*-LA-FP**	AACGTGAGCCTAGGGCGTGC*CCATGG*TCCCCGGTCCCCCCTTCT
***orf1*-LA-RP**	AACGTGAGCCTAGGGCGTGC*CCATGG*GCCGAACAGGTACCCCAGAC
***orf1*-RA-FP**	TTGGCTGACAATTGACATCT*GCTAGC*GGAATTCCTTGAACCCTA
***orf1*-RA-RP**	GTGGATCCGCACCCAAGCTT*GCTAGC*GACGTAGCTGATCAGTTC
**BafG-BamHI-FP**	TCGTGCCGGTTGGTA*GGATCC*AGGAGGGCGTGAGATGCACGTGTC
**BafG-KpnI-RP**	GGGCTGCAGGTCGACTCTAGA*GGTACC*TCACCCGGCGCGCATGTA
**Orf1-BamHI-FP**	TCGTGCCGGTTGGTA*GGATCC*AGGAGGCCGGGGAATGACACCGTC
**Orf1-KpnI-RP**	GGGCTGCAGGTCGACTCTAGA*GGTACC*CTAGGGTTCAAGGAATTC
**Spec-NdeI-FP**	TCATCTCGTTCTCCGCTCAT*CATATG*TGTAGGCTGGAGCTGCTTC
**Spec-SacI-RP**	AATACGAATGGCGAAAAGCC*GAGCTC*CTGACGCCGTTGGATACAC

Note: The italic underline litters indicate the restriction sites.

Table S2. The primers for construction of knock-out vectors and PCR confirmation of *S. lohii* mutants.

Primers	Sequence (5'-3')
***bafG*-KO-FP**	GCGGGGTCGATTCCGCCCGG
***bafG*-KO-RP**	ACATCCTCTCGGCCCACGAC
***orf1*-KO-FP**	CCGCGTTCGACCTCAAAGTC
***orf1*-KO-RP**	GTGCATTCACCGCCTTCGGA
**M13F-47**	CGCCAGGGTTTTCCCAGTCACGAC
**M13R-48**	AGCGGATAACAATTTCACACAGGA

Table S3. The primers for quantitative real-time PCR.

Primers	Sequence (5'-3')
***hrdB*-FP**	CCAAGAACCACCTCCTGGAG
***hrdB*-RP**	AGCCCTTGGTGTAGTCGAAC
***bafG*-FP**	CGAGAGGATGTGCCGGTGG
***bafG*-RP**	CTGGTGGGCAAGTTGATGCT
***orf1*-FP**	CCGTCAGCGACCTCTGAAGA
***orf1*-RP**	GACACCTTCCAGGATGCGGA
***bafAV*-FP**	ACTCGCCCCGTCGGAGGT
***bafAV*-RP**	GGGACAGGTTGGACTTCAGCG
